# The burden of meningococcal meningitis in the African Meningitis Belt, from 2009 to 2014: a trend analysis

**DOI:** 10.11604/pamj.2021.39.57.17629

**Published:** 2021-05-20

**Authors:** Anelisa Jaca, Alison Beriliy Wiyeh, Evanson Zondani Sambala, Charles Shey Wiysonge

**Affiliations:** 1 Cochrane South Africa, South African Medical Research Council, Francie van Zijl Drive, Parow Valley, 7501, South Africa,; 2 Centre for Evidence-based Health Care, Division of Epidemiology and Biostatistics, Department of Global Health, Faculty of Medicine and Health Sciences, Stellenbosch University, Cape Town, South Africa,; 3 Division of Epidemiology and Biostatistics, School of Public Health and Family Medicine, University of Cape Town, Cape Town, South Africa

**Keywords:** Emerging strains, MenAfriVac, meningitis, meningitis belt, serogroup A

## Abstract

**Introduction:**

Neisseria meningitides is the leading cause of meningitis in the African Meningitis Belt. The objective of this study was to conduct a trend analysis of the burden of meningococcal meningitis in the African Meningitis Belt countries from 2009 to 2014.

**Methods:**

secondary data on incidence and death cases were collected from the World Health Organization (WHO) and analyzed to determine the trends of meningitis in the African Meningitis Belt countries using Microsoft excel and Stata 14.

**Results:**

these data show unstable meningococcal meningitis outbreaks in the Meningitis Belt before and after the introduction of meningococcal A vaccine (MenAfriVac). The vaccine was introduced at different times in the different countries. E.g. it was introduced in 2010 across Burkina Faso, Mali and Niger while it was introduced from 2011 to 2016 in other countries through mass campaigns. Ever since the vaccine was introduced, there has been a decrease in the number of cases in the countries hence a reduction in the burden of the disease.

**Conclusion:**

after the introduction of the MenAfriVac, there has been a decline in the meningitis cases in Benin, Burkina Faso, Chad, Ghana, Niger and Nigeria while Sudan shows a decrease only in 2014.

## Introduction

**Meningococcal meningitis:** bacterial meningitis accounts for the majority of reported cases of meningitis, with the leading causes being Neisseria meningitidis, *Streptococcus pneumonia* and *Haemophilus influenza type b* (Hib) [1]. It causes inflammation of the meninges [2] typically resulting in high fever, headache, vomiting, neck stiffness and photophobia, though other symptoms can be present. It can be fatal if not treated and individuals who survive are left with permanent sequelae including hearing loss, neurologic impairments, seizures and paralysis [2-4]. Neisseria meningitidis is the leading cause of meningitis in the Meningitis Belt, a region consisting of 26 countries that stretch from Senegal in the west to Ethiopia in the east [5]. The occurrence, severity and spread of this disease is multifactorial; it is influenced by several host factors such as age and immune system function with children under five years and adolescents being the most severely affected. Environmental factors such as geographical location, climate and environment, and socioeconomic factors including urbanization level also influence the spread of the disease [6,7]. Neisseria meningitidis has 12 serogroups, with A, B, C, X, W and Y being responsible for the majority of this infection, worldwide [8]. Serogroup A was previously the main cause of 90% of cases of meningococcal meningitis in the Meningitis Belt [9], but recent outbreaks have been attributed to serogroups C and W [10]. In other regions (Europe, the Americas, and Australia), serogroups B, C, Y and W have been the main cause of the disease [10]. In sub-Saharan Africa, epidemics often occur in the dry season causing substantial morbidity and mortality in the population, further crumbling already deficient health systems in the region [11].

Historically, mass chemoprophylaxis with sulpha-based drugs was the main prevention strategy used against meningococcal meningitis. However, this intervention proved to be inefficient with the resurgence of epidemics and the emergence of resistant strains [11]. Thus in 2010, the meningococcal serogroup A conjugate vaccine (MenAfriVac) was introduced in a few countries of the African Meningitis Belt, where the disease burden was most significant [12]; however, all the 26 countries had received the vaccine by 2016 [13]. A high MenAfriVac coverage rate among individuals aged 1-29 years was reported; hence a decrease in the burden of disease is expected from the African Meningitis Belt [13]. However, due to several factors such as the changing pattern of the disease which has been attributed to climate change [14], there are still cases caused by C, W and X serogroups [15].

Though the last outbreak of meningococcal meningitis in the African Meningitis Belt was reported in 2015, there is a persistent risk of disease outbreaks. A better understanding of the causes of this disease is very important as this may contribute towards the preparedness of future outbreaks. Knowledge of the trends of meningococcal meningitis in the African Meningitis Belt region will facilitate the development and implementation of the most effective interventions for reducing the burden of the disease in these countries. In addition, understanding the trend of this disease will help with identifying priority settings for effective distribution of limited resources. The objective of this study is to conduct a trend analysis of the burden of meningococcal meningitis in the African Meningitis Belt countries. We compare trends of meningitis across the countries from 2009 to 2014.

## Methods

### WHO/Global Health Observatory (GHO) data and analysis

**Data source and collection:** we obtained data on the number of suspected cases and deaths caused by meningococcal meningitis in the Africa Meningitis Belt region from the WHO Global Health Observatory (GHO) in May 2018. As reported by the GHO, these data were last updated in April 2015. The GHO issues analytical reports on priority health issues such as mortality and burden of disease, health systems, environmental health, non-communicable diseases and infectious diseases [15]. Three types of data are collected on meningococcal meningitis: 1. Annual national data on the number of cases and deaths each year, obtained from officially reported data and published reports collected from regional offices twice a year. 2. Weekly data on the number of cases and deaths, obtained from national level data collected for countries in the Meningitis Belt. These data are reported weekly during the meningitis season and once every two weeks outside the meningitis season. 3. Local level data on specific outbreaks, obtained from WHO country offices and other organizations such as Médecins Sans Frontières (MSF).

**Case definitions:** our outcome measures were the number of suspected meningitis cases and deaths reported. As per standard case definitions for meningococcal meningitis, a suspected case of acute meningitis is defined as a sudden onset of fever (>38.5 °C rectal or 38.0 °C axillary) with neck stiffness. In children under one year of age, a suspected case of meningitis is defined as fever accompanied by a bulging fontanelle [16]. To confirm the diagnosis of meningococcal meningitis, N. meningitides must be detected in the cerebrospinal fluid (CSF) using latex agglutination or cultured from CSF or blood. A suspected meningitis death is defined as the number of deaths amongst suspected cases of meningococcal meningitis [16].

**Data analysis:** the data collected from WHO/GHO website were entered into a table to form the final data set from 2009 to 2014. The years in which the MenAfriVac was introduced and number of suspected cases (from 2009 to 2014) for each country was indicated (Table 1).

**Table 1:** WHO/GHO data showing meningococcal meningitis suspected cases (2009 to 2014) and the years of MenAfriVac introduction in the Meningitis Belt countries

		Number of suspected cases from 2009 to 2014					
**Country**	**Year of vaccine introduction**	**2009**	**2010**	**2011**	**2012**	**2013**	**2014**
Benin	2012	416	323	269	1165	833	711
Burkina Faso	2010	4723	6732	3875	6957	2917	3476
Cameroon	2011	1001	835	2548	542	1010	1156
Central African Republic	2016	289	477	361	266	210	169
Chad	2011	1460	3228	5960	3874	371	235
Côte d'Ivoire	2014	284	146	141	500	255	196
Democratic Republic of the Congo	2016	4842	8276	5176	10141	9339	10109
Ethiopia	2013	114	No data	229	150	No data	1744
Gambia	Not reported	No data	No data	No data	200	248	214
Ghana	2012	302	1164	773	739	454	448
Guinea	2015	81	84	No data	196	480	582
Mali	2010	335	482	430	688	358	327
Mauritania	2014	No data	No data	No data	41	14	1
Niger	2010	13449	2908	1214	314	311	327
Nigeria	2011	56128	4983	1165	1206	871	1175
Senegal	2012	No data	No data	No data	894	379	102
South Sudan	2016	No data	No data	No data	No data	259	111
Sudan	2012	No data	2240	No data	524	1110	207
Togo	2014	350	460	440	408	266	351

## Results

**African meningitis belt region:** the suspected cases and mortality cases of meningococcal meningitis were available and accessed from the GHO for 14 out of 26 Meningitis Belt countries, i.e., Benin, Cameroon, Chad, Ethiopia, Gambia, Ghana, Guinea, Mali, Mauritania, Niger, Nigeria, Senegal, Sudan and Togo [15] from 2009 to 2014 (Table 1, Table 2). Data on incidence and death cases were not available in some years for various countries, namely, Ethiopia (2010 and 2013), Gambia (2009 to 2011), Guinea (2011), Mauritania (2009 to 2011), Senegal (2012 to 2014) and South Sudan (2011 to 2014). Our analysis indicates a high number of meningitis cases in the Meningitis Belt countries before the MenAfriVac was introduced (Table 1). These results show that only three countries (Burkina Faso, Mali and Niger) introduced the MenAfriVac among the African Meningitis Belt countries in December 2010 (Table 1). The data presented between 2009 and 2010 indicate a high number of meningitis cases in Burkina Faso while there was a decline of cases in 2011, that is, after the MenAfriVac was introduced. Interestingly, in 2012, there was another increase of meningitis cases in Burkina Faso. Mali shows quite a minimum number of cases between 2009 and 2011 while there was an increase in the number of cases in 2012. Moreover, Niger had the highest number of cases between the years 2009 and 2010, while there was a decrease after the introduction of MenAfriVac (Table 1).

**Table 2:** WHO/GHO data showing meningococcal meningitis suspected death cases (2009 to 2014) and the years of MenAfriVac introduction in the Meningitis Belt countries

		Number of suspected death cases from 2009 to 2014					
Country	Year of vaccine introduction	2009	2010	2011	2012	2013	2014
Benin	2012	64	54	50	112	82	88
Burkina Faso	2010	629	949	588	709	339	353
Cameroon	2011	122	71	165	64	68	60
Central African Republic	2016	48	106	46	31	31	41
Chad	2011	152	248	270	163	40	22
Côte d'Ivoire	2014	46	28	25	59	34	25
Democratic Republic of the Congo	2016	514	926	509	1011	859	994
Ethiopia	2013	20	No data	9	0	No data	52
Gambia	Not reported	No data	No data	No data	9	34	38
Ghana	2012	63	128	57	75	41	17
Guinea	2015	8	11	No data	13	51	55
Mali	2010	28	34	17	12	7	4
Mauritania	2014	No data	No data	No data	6	5	0
Niger	2010	558	251	145	56	44	40
Nigeria	2011	2488	337	62	74	47	81
Senegal	2012	No data	No data	No data	28	10	12
South Sudan	2016	No data	No data	No data	No data	14	2
Sudan	2012	No data	98	No data	25	49	5
Togo	2014	47	94	43	35	22	14

Furthermore, among the three countries (i.e. Cameroon, Chad and Nigeria) that introduced the vaccine in 2011, Nigeria has the highest number of meningitis cases between 2009 and 2010. The number of cases started decreasing from the year that MenAfriVac was introduced with unstable incidences from 2012 to 2014. Chad also showed the highest number of cases in 2011 where cases decreased from 2012 to 2014 (Table 1). Concerning Benin, Ghana, Senegal and Sudan, the vaccine was introduced in 2012. Our results therefore indicate a decline in the number of cases in Benin, Ghana and Senegal between from 2013 to 2014 while Sudan shows a decrease only in 2014 (Table 1). There were no data for Ethiopia in 2013 hence it was not possible to assess whether the vaccine was effective or not after it was introduced. There were also no data available after the vaccine was introduced in Cote D´Ivoire, Mauritania, Togo, Guinea, CAR, DRC and South Sudan hence we could not determine how effective the vaccine in those countries (Table 1).

## Discussion

**MenAfriVac in the African meningitis belt:** prevention of meningococcal meningitis remains a global challenge with strains of group A meningococcus causing unstable outbreaks, predominantly in the African Meningitis Belt [3,17]. In this paper, we highlight variations in trends of meningitis caused by serogroup A meningoccocci in the African Meningitis Belt countries from 2009 to 2014. There has been high incidence and mortality cases of the disease in the countries that encompass the Belt, namely, Benin, Burkina Faso, Cote d´Ivoire, Democratic Republic of Congo (DRC), Ghana, Niger, Nigeria, Senegal, Sudan and Togo with Nigeria, Niger, DRC and Burkina Faso being the most affected countries (Table 1, Table 2). In 2009, reported cases were as high as 13449 and 56128 in Niger and Nigeria, respectively. High mortality cases in the two countries, i.e. 558 and 2488 deaths in Niger and Nigeria were also seen (Table 2).

Furthermore, it is interesting to note that although there was a high number of cases in Burkina Faso (6957 cases) and DRC (10141 cases) in the year 2012 (Table 1), mortality cases were not as high as would be expected (Table 2). High incidence and mortality cases in the Meningitis Belt countries could be attributed to various factors. The epidemic of meningitis in countries like Nigeria could be associated with several factors, i.e. the primary reason most likely being household crowding, active and passive smoking [18,19]. Furthermore, various risk factors including low socioeconomic status, ethnicity and compromised immunity may predispose individuals to meningococcal meningitis [20]. Some studies showed that individuals from low socioeconomic status were at higher risk of meningococcal disease than those with high socioeconomic status [18]. Other factors including lack of proper awareness of meningococcal meningitis, suggest that it is difficult to reach other communities. Due to lack of proper awareness, the implications and symptoms of the disease are not always known; therefore, cases may not be reported and treated in time [18].

In response to high cases of this disease, there have been interventions introduced to alleviate the outbreak of the disease. These include enhanced surveillance for meningococcal disease [3], mass vaccination campaigns and introduction of MenAfriVac in the various countries [20]. Vaccination campaigns substantially reduce the burden of meningococcal meningitis; these campaigns are set off when an outbreak exceeds an epidemic threshold [21,22]. While these vaccination campaigns were established to alleviate the burden of meningococcal meningitis in many settings, their implementation is limited, due to delays in the diagnosis and reporting of meningitis cases [22]. Other factors affecting implementation of these campaigns involve vaccine affordability, availability and supply. Administering affordable meningococcal vaccines offers the opportunity for more successful control and potential eradication of meningitis in the Meningitis Belt [23].

Following the introduction of the MenAfriVac in the African Meningitis Belt countries from 2010, there has been a decrease in the number of cases in several countries including Benin, Burkina Faso, Chad, Ghana, Niger, Nigeria and Sudan. The decrease in the number of cases in these countries suggests that the use of MenAfriVac was very effective. This vaccine was administered to individuals aged 1 to 29 years throughout Burkina Faso and in some areas of Mali and Niger in December 2010, which decreased incidence and death rates. Worthy of note is the fact that there have been high cases of the disease in Burkina Faso and Mali even after the vaccine was introduced (Table 1). Whilst the meningitis vaccine was introduced in 2010 in Burkina Faso and Mali, there were high cases of meningitis in 2012 than before. The increased meningitis incidence and death cases even after the vaccine introduction could be attributed to factors including other bacterial strains (W135, C and X) responsible for causing meningococcal meningitis in the African Meningitis Belt [4,24].

Although serogroup A has decreased in countries that have implemented vaccination campaigns, other serogroups, including W and X, have the potential to cause epidemics. Hence more meningitis cases caused by serogroup W were reported in Burkina Faso in 2012 than in 2011 [25]. Concerning Sudan, it was not possible to determine whether there was an increase or decrease in cases of the disease since there were no data reported in 2012. Moreover, we cannot assess the effect of MenAfriVac on meningitis cases in countries where the vaccine was introduced from 2014 since there are no incidence data available from that year.

**Limitations:** many countries in the Meningitis Belt have weak disease surveillance and response systems and continue to face challenges in accurately diagnosing and reporting this disease. The case definitions for meningococcal meningitis across and within countries has not been harmonized. While some countries only report laboratory confirmed cases, others also report suspected cases. These variations in reporting make it difficult to compare disease burden across countries and could lead to a misrepresentation of the actual disease burden [26].

## Conclusion

Meningitis remains a public health concern in the African Meningitis Belt [27]. Emerging strains of *Neisseria meningitides* and changing environmental conditions have been reported to be a cause of epidemic meningitis in this region. However, after the introduction of meningitis vaccines, there has been a decline in the trend of cases in the Meningitis Belt over the years. This decline has been attributed to the mass vaccination campaign after the 2010 epidemic [28]. This vaccination campaign includes an introduction of a monovalent serogroup A meningococcal conjugate vaccine (MenAfriVac) in 16 of the Meningitis Belt countries among individuals aged 1 to 29 years. Therefore, epidemics due to serogroup A have been reduced hence recent epidemics have been primarily due to serogroups C and W. It is important to note that the study results should be interpreted considering some limitations. One limitation is the use of secondary sources of data, which may have caused inconsistencies and introduced bias toward estimating and reporting meningitis cases.

### What is known about this topic

- Neisseria meningitidis has 12 serogroups, with A, B, C, X, W and Y being responsible for most of this infection worldwide;
- Serogroup A was previously the main cause of 90% of cases of meningococcal meningitis in the Meningitis Belt but recent outbreaks have been due to serogroups C and W;
- Among the African Meningitis Belt countries, Nigeria, Niger, DRC and Burkina Faso are the most affected countries.

### What this study adds

- Although serogroup A has decreased in countries that have implemented vaccination campaigns, other serogroups, including W and X, have the potential to cause epidemics;
- Large outbreaks of meningitis in countries like Nigeria could be caused by factors like household crowding and active and passive smoking, low socioeconomic status, ethnicity and compromised immunity;
- Meningococcal serogroup A conjugate vaccine (MenAfriVac) was introduced in a few countries of the African Meningitis Belt, where the disease burden was most significant.
